# The impact of delayed access to COVID-19 vaccines in low- and lower-middle-income countries

**DOI:** 10.3389/fpubh.2022.1087138

**Published:** 2023-01-12

**Authors:** Brenice Duroseau, Nodar Kipshidze, Rupali Jayant Limaye

**Affiliations:** ^1^Center for Infectious Disease and Nursing Innovation, Johns Hopkins University School of Nursing, Baltimore, MD, United States; ^2^Department of Epidemiology, Mailman School of Public Health, Columbia University, New York, NY, United States; ^3^Department of International Health, Johns Hopkins Bloomberg School of Public Health, Baltimore, MD, United States; ^4^International Vaccine Access Center, Johns Hopkins Bloomberg School of Public Health, Baltimore, MD, United States

**Keywords:** COVID-19, vaccine equity, LMICs, global health, immunization (vaccination)

## Abstract

**Introduction:**

A majority of low-income (LIC) and lower-middle-income countries (LMIC) were unable to achieve at least 10% population coverage during initial vaccine rollouts, despite the rapid development of the coronavirus disease 2019 (COVID-19) vaccines. Nearly three years into this pandemic, evaluating the impact of inequities in vaccine access, uptake, and availability is long overdue. We hypothesized that a delay in receiving COVID-19 vaccines was associated with an increased toll on cumulative cases and mortality. Furthermore, this relationship was modified by the size of a country's economy.

**Methods:**

We performed an ecological study assessing these relationships, in which a country's economic standing was assessed by world bank income classification, gross domestic product based on the purchasing power parity (GDP PPP) per capita category, and crude GDP PPP.

**Results:**

Countries with the smallest economies reported first vaccination much later than larger economies on all three rankings, as much as 100 days longer. Among low-income countries, a one-day increase until the first vaccination was associated with a 1.92% (95% CI: 0.100, 3.87) increase in cumulative cases when compared to high-income countries (*p* = 0.0395) when adjusting for population size, median age, and testing data availability. Similarly, among the lowest GDP PPP countries a one-day increase until the first vaccination was associated with a 2.73% (95% CI: 0.100, 5.44) increase in cumulative cases when compared to the highest GDP PPP countries (*p* = 0.0415). When modeling cumulative mortality, effects in the same direction and magnitude were observed, albeit statistically non-significant.

**Conclusion:**

Economic standing modified the effects of delayed access to COVID-19 vaccination on cumulative cases and mortality, in which LMICs tended to fare worse in outcomes than high-income countries despite the eventual rollout of vaccines. These findings highlight the importance of prioritizing equitable and timely access to COVID-19 vaccines across all countries, irrespective of economic size. Future studies should examine the impacts that vaccine inequities had on local transmission dynamics.

## 1. Introduction

Accelerated development, testing, and distribution of coronavirus disease 2019 (COVID-19) vaccines have proven paramount in reducing worldwide morbidity and mortality. In the first year of vaccination, an estimated 14.4 million deaths were averted globally because of COVID-19 vaccination (1). While the impact of COVID-19 vaccines cannot be understated, especially after quantifying the reduction in disease burden and mortality, significant inequities persist in vaccine access, uptake, and availability globally (1–5).

The COVID-19 vaccines have proven effective in preventing severe COVID-19, however, as of October 2022, only 25% of the population living in low-income countries have received at least one dose of a vaccine (6). This statistic is in contrast to high-income countries (HIC), where 72% of the population has been vaccinated with at least one dose (6). The countries with the smallest economies, that is, those with the lowest gross domestic product (GDP), have been lagging in COVID-19 vaccination rollout, with many low- and lower-middle-income countries (LMIC) achieving <10% population coverage in the initial rollouts of COVID-19 vaccines through Spring 2021 (7). Delays in vaccine rollout, caused primarily by inequities and inequalities (7), could have devastating effects on low-income countries.

Early in the pandemic, there was a push to better understand the toll of the pandemic in the most resource-poor countries. Both LIC and LMICs already carry a burden of higher human immunodeficiency virus/acquired immunodeficiency syndrome (HIV/AIDS) and tuberculosis (TB) incidence in comparison to the rest of the world, though it is estimated these nations also have far fewer ICU beds and hospital beds in comparison to HICs (8). Initial estimates indicated that HICs were disproportionately impacted by the pandemic, with higher reported case counts and deaths. However, LIC/LMICs have had less testing capacity for COVID-19, and therefore not all COVID-19 fatalities were attributed as such, with excess mortality per capita estimated to be the highest among LIC/LMICs when compared to HICs (9). It has also been reported that many deaths in poorer nations occur within rural communities where deaths do not get ascertained in official statistics (10).

Beyond the impact on health outcomes, the economic toll among LICs and LMICs has been unprecedented. In the second quarter of 2020, it was estimated that LICs experienced as much as a 13.4% loss in working hours and a 29% loss among LMICs, in comparison to the 15.8% loss among HICs (11). In the first half of 2021, inflation was on the rise in low-income countries and has continued to rise sharply given the recent Russian invasion of Ukraine, leading to high food prices and acute food shortages in already fragile economies (12). A strong association has been found between national COVID-19 vaccination rates and individual-level financial impacts, in which countries that have the lowest vaccination rates reported the highest individual-level financial impact. The odds of being financially impacted among LICs was 16.88 (95%CI: 14.69, 19.39) times the odds of HICs (13). Larger proportions of workers in LICs make a living through informal jobs that require mobility and travel, compared to workers in HICs or upper-middle-income countries (UMICs) who have at home remote options. In addition, contact patterns vary by country, with HIC/UMICs reporting decreased contact rates in elderly groups with younger age groups. In contrast, LIC/LMICs demonstrated no decline in contact patterns, in which more vulnerable groups maintained similar contact patterns as younger individuals (14). Finally, lockdown measures impacted LICs and LMICs differently than UMICs and HICs which have more stable economies and additional resources, which afforded government support *via* welfare protection networks, pandemic stipends, continued healthcare service provisions, etc. (15). Since Fall 2022, the same trends persist with many low-income countries barely reaching 20% population coverage for primary COVID-19 vaccination series and substantially less for booster doses (16). Even more troubling, only ~37% of healthcare workers across low-income countries have completed a primary vaccination series (17). Since the inception of the COVID-19 vaccines global access initiative (COVAX) in April 2020, countries, or unions with the largest economies (i.e., the United States and EU) have donated <50% of their announced contributions (18). During the June 2022 COVAX allocation review session, it was reported that 182 million doses of unused vaccines were planned to be destroyed across the US and the EU (19). Health outcomes and public health capacity, especially during the pandemic, are heavily driven by economics and politics (20–22). Countries with smaller economies cannot afford, are priced out, or are charged more than some high-income nations. It was found that countries such as Uganda or South Africa were paying $7 and 5.25 per dose, respectively, in comparison to some European countries that were paying $3.50 per dose (23). Several LIC/LMIC attempted to enact the Trade Related Aspects of Intellectual Property Rights (TRIPS) agreements to waive intellectual property (IP) rights to afford these life-saving vaccines, however, these requests were denied by the G7. Purchasing power, IPs, and initial negotiations served as financial barriers that contributed to the higher price point of COVID-19 vaccines in LICs and LMICs. Therefore, the donation of vaccines has proven important, however, the fulfillment of donation promises by HICs has been disappointing in practice. In addition to the affordability of the vaccines or the purchasing power, LMICs continue to face limitations in testing capacity, effective contact tracing, and basic medical care.

In light of the apparent disparities, we sought to: (1) quantify the time to the first vaccine in each country using the available data; (2) determine whether the time to the first vaccine varied by economic standing or economic size; and (3) determine whether the time to the first vaccine had an effect on COVID-19 cases and mortality. We hypothesized that countries with the smallest economies would not have access to vaccines as early as those with the largest economies and that this delayed access to vaccines would have a disproportionately negative effect on the COVID-19 outcomes in these countries.

## 2. Methods

### 2.1. Study design and data sources

This study was an ecological study assessing the differences in the time of access to COVID-19 vaccines and COVID-19 outcomes. The unit of analysis was individual countries. All data were retrieved from publicly available data sources (Supplementary Table 1). Briefly, data were sourced from the World Health Organization (WHO) COVID-19 data dashboard and Our World in Data (24). All data were downloaded between July 2022 and October 2022.

### 2.2. COVID-19 cumulative cases and mortality

Cumulative cases and mortality were taken from the official counts found in the WHO COVID-19 data dashboard. The total number of cases and deaths was taken from the start of the pandemic through 31 May 2021. We considered a longer date range, however, given the then-soon introduction of the Delta variant of concern (VOC), case counts and mortality may have been confounded by the circulation of newer VOCs (25, 26).

### 2.3. Time to first vaccination

The time to the first vaccination was calculated as the difference in days between the WHO-reported first vaccination for each country and the first-ever confirmed COVID-19 case, globally (31 December 2019). Time to vaccination was compared across economic groups using one-way ANOVA or Pearson correlation, as applicable.

### 2.4. Effect modification of economic standing

In our casual model (Supplementary Figures 1A, B), income classification was considered as a possible effect modifier in the relationship between the timing of vaccine access and COVID-19 outcomes. It was hypothesized that countries with a few economic advantages (i.e., low- or lower-middle-income countries) would typically gain access to vaccines much later than high-income countries. This association was assessed using three different operationalizations of this hypothesized interaction. First, countries were grouped according to 2021 world bank-defined income classifications: low-income, lower-middle-income, upper-middle-income, and high-income. Second, the log-transformed gross domestic product based on the purchasing power parity (GDP PPP) per capita was binned into five categories: <7.47, 7.78–8.48, 8.49–9.47, 9.48–10.46, and >10.46. Third, the crude log-transformed GDP was assessed as a continuous variable. Each operationalization was run in separate models for both cumulative COVID-19 cases and COVID-19 mortality.

### 2.5. Confounders

Several covariates were considered in their confounding effect between time to vaccine and COVID-19 outcomes. In modeling the impacts of COVID-19, case counts, stringency index, testing rates, median age, and population size were all initially considered to confound the main causal relationship of interest (Supplementary Figure 1A). However, the stringency index did not contribute significantly to the model and was therefore removed. Testing rates were operationalized by the number of tests per capita. However, it was found that many countries did not have testing data available. The odds of no data availability among low-income countries was 9.917 (3.202, 30.711) times the odds of no data availability among high-income countries (*p* < 0.0001). Similarly, the odds of no data availability among the lowest GDP category countries was 15.862 (3.214, 78.288) times the odds of no data availability among the highest GDP countries (*p* = 0.0007). Finally, on average, the difference between countries with and without testing data availability differed by −2.144 (95% CI: −3.095, −1.194) units when comparing across GDP PPP (*p* < 0.0001). Therefore, to avoid restricting the analysis to a small subset of countries, the reliability of reported case counts or mortality was operationalized as the availability of testing data (yes/no). Models were run with and without accounting for testing rates; however, the models without testing rates are presented here and the models with testing rates can be found in the Supplementary Table 2. Population size was found to be extremely skewed, and log-transformed, improving the linear relationship between this covariate and each respective outcome. In the modeling of COVID-19 mortality, in addition to the covariates considered in the case counts model, the proportion of the population more than 65 years old, the proportion of the population more than 75 years old, cardiovascular mortality rate, hospital beds per capita, and life expectancy were all considered as possible confounders (Supplementary Figure 1B). Ultimately, these covariates were not found to contribute meaningfully to the model and were therefore not included, leaving no differences in the covariates used in the modeling of mortality and of cases.

### 2.6. Data analysis

Multiple linear regression was used to assess the relationship between the time to the first vaccine and COVID-19 cumulative cases and mortality. All model coefficients, 95% confidence intervals, and fit statistics were estimated using an ordinary least squares regression model (OLS). Categorical variables and interaction terms were dummy coded as appropriate. The same models were run again using a generalized linear model (GLM) to estimate additional fit statistics: AIC and BIC scores. Model estimates, model diagnostics, and descriptive statistics were performed in SAS (Version 9.4; SAS Institute). Visualizations of both categorical by continuous and continuous by continuous interactions were created using the *emmeans* package in R (version 4.0.03; R Foundation).

## 3. Results

### 3.1. Time to the first vaccine

**Figure 1:**
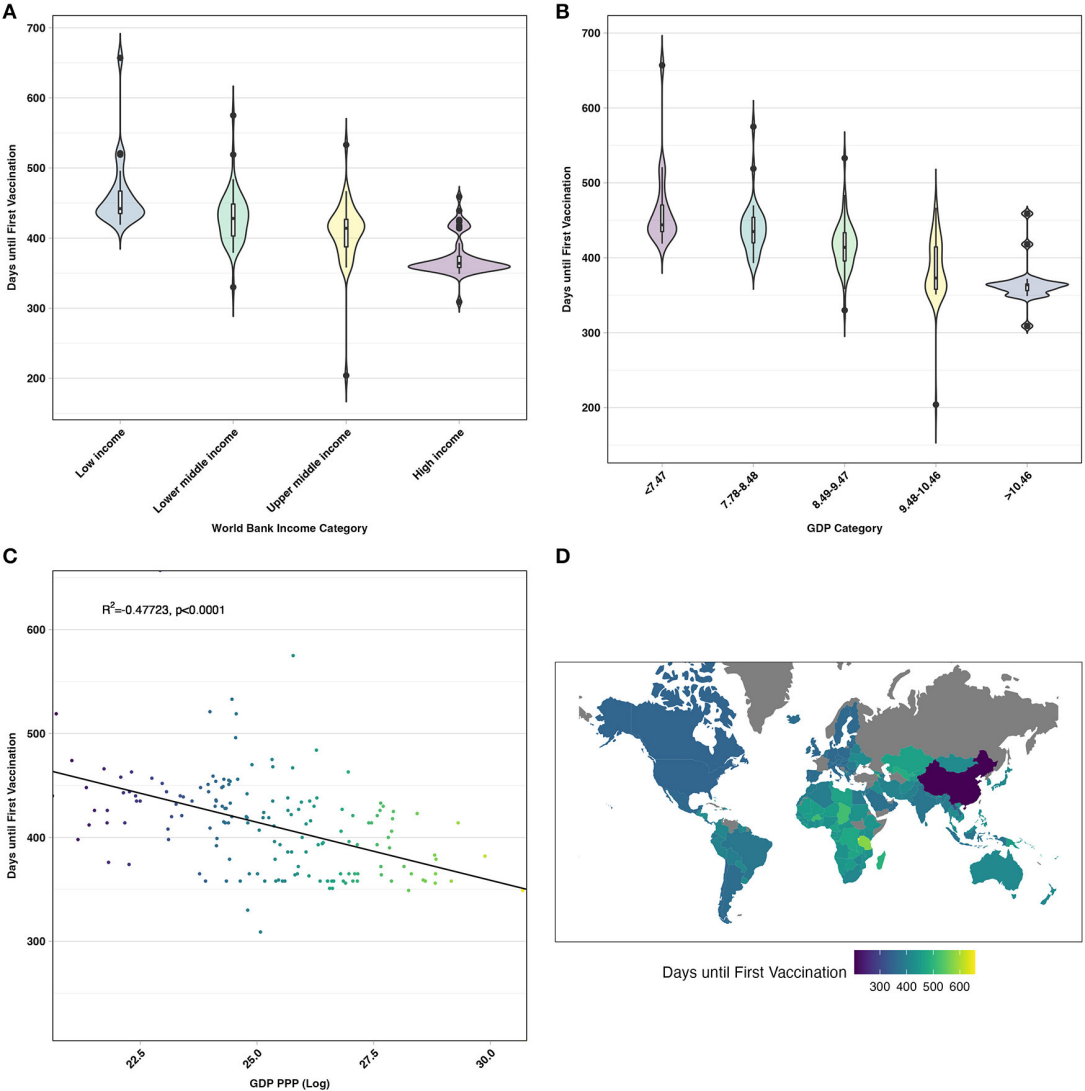
**(A)** Comparing the distribution of days until the first vaccination between world bank income categories. **(B)** Comparing the distribution of days until the first vaccination by log-GDP PPP per capita category. **(C)** Comparing days until first vaccination and log-crude GDP PPP. **(D)** Choropleth of days until the first vaccination by country, with lighter shades indicating a longer time to report the first COVID-19 vaccines.

The time to the first vaccination since the initial COVID-19 reported case (31 December 2019) was compared among countries by world bank income classifications (Figure 1A), log-transformed GDP PPP per capita categories (Figure 1B), and log-transformed GDP PPP (Figure 1C). Low-income countries on average lagged behind by 89.533 (95% CI: 50.829, 128.238) days until first vaccination, when compared to high-income countries (*p* < 0.0001) (Supplementary Table 3). Similarly, the lowest GDP PPP category lagged behind the highest GDP category by 100.034 (95% CI: 50.058, 150.01) days until the first vaccination (*p* < 0.0001) (Supplementary Table 4). With increasing world bank income classifications and GDP PPP categories, the time to vaccination decreased (Figures 1B, C, Supplementary Tables 3, 4). When comparing time to the first vaccination by GDP PPP as a continuous variable (Figure 1C), there was a moderate negative correlation (R^2^ = 0.47723, *p* < 0.0001) with higher GDP PPP countries averaging a shorter time to the first vaccination in comparison to lower GDP PPP countries. Figure 1D demonstrates the time until the first vaccination by country.

### 3.2. Impact of delayed access to vaccines on COVID-19 outcomes

#### 3.2.1. Cumulative cases

The impact of delayed access to vaccines on cumulative case counts was modeled using a linear regression model. Log-transformed cumulative case counts of each country from the beginning of the COVID-19 pandemic through 31 May 2021 were modeled as the outcome. Delayed access was operationalized as days to first reported vaccinations. The interaction between economic classifications (world bank income category, GDP PPP per capita category, and GDP PPP) and time to the first vaccine was included in the full models. Testing data availability, 2021 log-transformed population estimates, and median age were included as possible confounders.

When controlling for all confounders, among low-income countries, a 1-day increase until the first vaccination was associated with a 1.92% (95% CI: 0.100, 3.87) increase in cumulative cases when compared to high-income countries (*p* = 0.0395) (Table 1). Among lower-middle-income countries, a one-day increase until the first vaccination was associated with a 1.11% (95% CI: −0.70, 2.94) increase in cumulative cases when compared to high-income countries (*p* = 0.2351). Finally, among upper-middle-income countries, a 1-day increase until the first vaccination was associated with a 3.46% (95% CI: 1.71, 5.23) increase in cumulative cases when compared to high-income countries (*p* = 0.0001). Similar trends were observed using other economic classifications. In the adjusted model, among the lowest GDP PPP countries, a 1-day increase until the first vaccination was associated with a 2.73% (95% CI: 0.100, 5.44) increase in cumulative cases when compared to the highest GDP PPP countries (*p* = 0.0415). Finally, the interaction between GDP PPP (as continuous) and days to vaccine was significant (*p* = 0.0377).

**Table 1:** Assessing the interaction between economic classification on the associations between time to the first vaccine and cumulative cases.

**Parameter**	**Cumulative cases**					
	**Crude model**		Full model without interaction^*^		Full model with interaction^*^	
	**β (95% CI)**	**P-value**	**β (95% CI)**	**P-value**	**β (95% CI)**	P-values^***^
**I. World bank income classifications**						
Days to vaccine	−0.022 (−0.029, −0.015)	< 0.0001	−0.006 (−0.012, 0)	0.0631	−0.026 (−0.04, −0.012)	0.0005
**Income classifications**						
Low income			−0.871 (−2.226, 0.484)	0.2062	−8.275 (−16.038, −0.511)	0.0369
Lower middle income			−0.186 (−1.168, 0.796)	0.7087	−3.905 (−11.022, 3.212)	0.2801
Upper middle income			0.483 (−0.256, 1.222)	0.1989	−12.787 (−19.402, −6.172)	0.0002
High income			—	—	—	—
**Income classifications x days to vaccine**						
Low income					0.019 (0.001, 0.038)	0.0395
Lower middle income					0.011 (−0.007, 0.029)	0.2351
Upper middle income					0.034 (0.017, 0.051)	0.0001
High income			—	—	—	—
**II. GDP PPP** ^**^						
Days to vaccine			−0.006 (−0.012, 0.001)	0.091	−0.065 (−0.121, −0.009)	0.0239
GDP PPP			0.308 (−0.135, 0.751)	0.1714	−0.672 (−1.695, 0.351)	0.1962
GDP PPP x days to vaccine					0.002 (0, 0.004)	0.0377
**III. GDP PPP per capita**						
Days to vaccine			−0.007 (−0.013, 0)	0.0464	−0.033 (−0.056, −0.01)	0.0048
**GDP** ^**^						
< 7.47			−0.27 (−1.648, 1.107)	0.6991	−10.103 (−20.287, 0.082)	0.0518
7.78–8.48			0.393 (−0.802, 1.587)	0.517	−0.035 (−10.884, 10.814)	0.995
8.49–9.47			0.699 (−0.232, 1.629)	0.14	−11.037 (−20.924, −1.15)	0.0289
9.48–10.46			0.599 (−0.19, 1.388)	0.1359	−12.679 (−21.794, −3.565)	0.0067
>10.46 (Reference)			—	—	—	—
**Days to vaccine x GDP** ^**^						
< 7.73					0.027 (0.001, 0.053)	0.0415
7.74–8.97					0.005 (−0.022, 0.033)	0.7045
8.98–10.21					0.031 (0.005, 0.057)	0.0181
9.48–10.46					0.036 (0.011, 0.06)	0.0046
> 10.21 (Reference)			—	—	—	—

GDP, gross domestic product; PPP, purchasing power parity.

* Adjusted for 2021 population size, median age, and testing data availability (yes/no).

** Log-transformed.

*** Modeled using multiple linear regression, p-values for t-test unless otherwise indicated.

**Figure 2:**
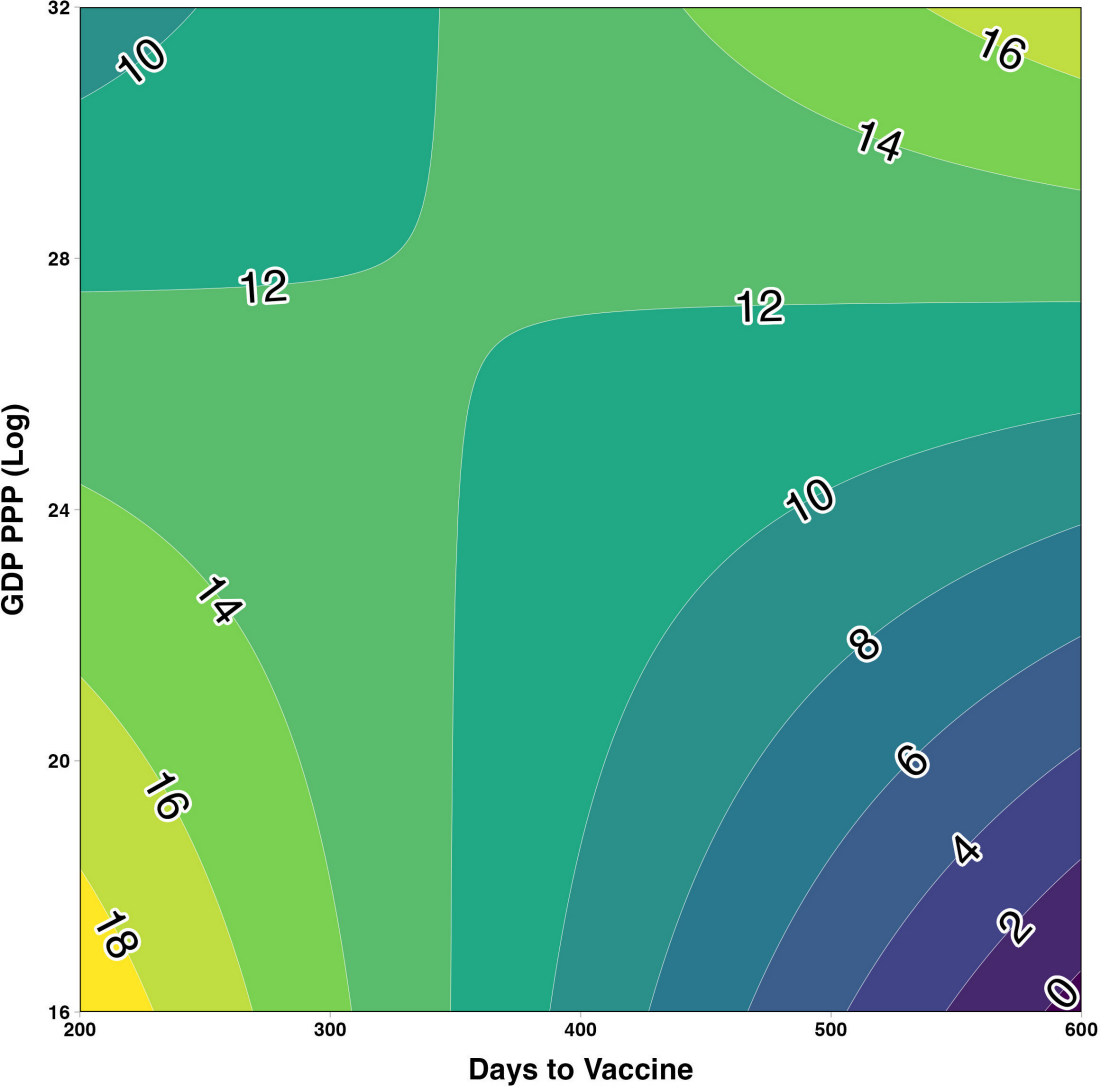
Contour plot assessing the two-way interaction between log-GDP PPP and days to first COVID-19 vaccine on cumulative cases estimated using multiple linear regression and adjusted for 2021 population size, median age, and testing data availability (yes/no).

**Figure 3:**
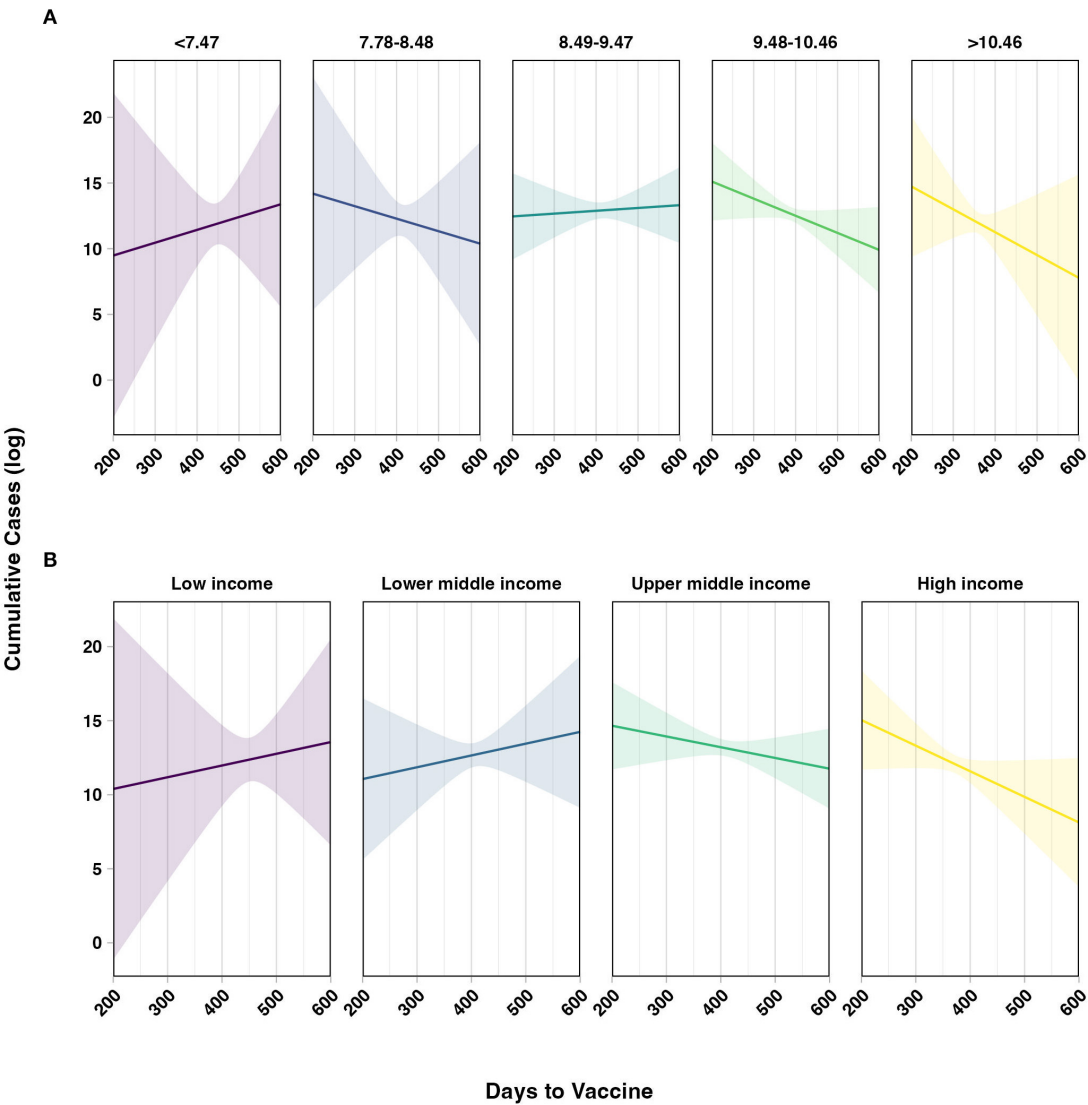
Assessing the two-way interaction between economic size and days to first COVID-19 vaccine on estimated log-cumulative cases. **(A)** Economic size operationalized as world bank income classification. **(B)** Economic size operationalized as categorical log-GDP PPP per capita. Both models adjusted for 2021 population size, median age, and testing data availability (yes/no). Shaded bands indicate 95% prediction intervals.

Predicted values were estimated and plotted in a contour plot with contours depicting predicted log-cumulative cases (Figure 2). It was predicted that among countries reporting the first vaccine under 350 days, the highest log cumulative cases were among the lowest GDP countries. Conversely, among countries reporting the first vaccine over 350 days, the highest predicted log cumulative cases were among the highest GDP countries vs. low GDP countries which trend downward as days to vaccine increased. Similar interaction plots were visualized for the two other economic classifications. Among the lowest GDP PPP category, the predicted log-cumulative cases trended upward as days to vaccine increased (Figure 3A). In comparison, the highest GDP PPP category trended downward, in which countries that waited longer until the first vaccine reported lower log-cumulative cases. A similar trend was seen with predicted trends compared across world bank income classifications (Figure 3B). Low- and lower-middle-income countries trend upward in log cumulative cases as days to first vaccine increased, whereas upper-middle-income and high-income countries trended toward lower log-cumulative cases.

#### 3.2.2. Cumulative deaths

The impact of delayed vaccination on cumulative mortality was modeled by adjusting for the same confounders (Supplementary Table 5). Unlike modeling cumulative cases, the effect modification of country-level economic classification was less significant for cumulative mortality in the different economic models. However, the direction and magnitude of the interaction were similar to the models for cumulative cases. Among low-, lower-middle-, and upper-middle-income countries, respectively, a 1-day increase in first vaccination was associated with a positive percentage increase in cumulative mortality. Similarly, among the lowest GDP PPP category, a 1-day increase to the first vaccination was associated with an increase in cumulative mortality when compared to high-income countries. Finally, when holding GDP PPP (continuous) constant, an increase in days to the first vaccination was associated with an increase in cumulative mortality. Finally, the interaction terms for cumulative deaths were also plotted to better understand the predicted trends (Supplementary Figures 2A, B). Unsurprisingly, the prediction intervals were much larger for the mortality models given the insignificant findings in comparison to the case models. A similar trend was observed for the two-way interaction when predicting cumulative mortality (Supplementary Figure 3).

## 4. Discussion

In this ecological study, the effects of delayed access to COVID-19 vaccination on COVID-19 cumulative cases and mortality were modified by the economic standing of the respective country. Countries ranking as low-income, lowest GDP PPP per capita, or crude GDP tended to be far poor with COVID-19 outcomes when compared to high-income countries or countries with larger economies. These trends were not immediately apparent, as simple correlations revealed that delayed vaccination was negatively associated with COVID-19 cumulative cases and mortality. In other words, countries with the longest waiting period to receive COVID-19 vaccines tended to report the least number of cases or deaths. On average, countries ranking the lowest by varying economic classifications were found to have the longest delays when compared to the highest income ranking countries. However, when adjusting for confounding and the effect modification of economic standing, the direction of these effects was found to be reversed. This study illuminates the significance of accounting for the size of countries' economies when evaluating the impacts of the COVID-19 pandemic. Additionally, these findings highlight the importance of prioritizing equitable, timely, and appropriate access to COVID-19 vaccines in countries with the smallest economies (27, 28).

Timely access to vaccines is critical in reducing the impact of emerging infectious diseases. Early allocation strategies by the WHO called for the equitable distribution of vaccines upon approval by relevant regulatory agencies (29). However, high-income countries reported, on average, a sooner time to the first vaccine rollout. The distribution of time among HICs was also less spread out, with much smaller tails in comparison to other countries. Several early studies examined within-country and between-country allocation strategies that prioritized distribution based on the older age groups or ongoing incidence (29–31), which may have contributed to inequitable distributions. Typically, LICs/LMICs have younger populations in comparison to HICs (32). Our study revealed that, on average, LICs were faced with the longest time to the first vaccine rollout, with much longer tails in the upper bound. Vaccine hesitancy or barriers to uptake among LMICs are often cited as a justification for the inequitable distribution of COVID-19 vaccines. Arce and colleagues studied the willingness of COVID-19 vaccine uptake in 10 LMICs across Asia, Africa, and South America (33). Willingness to take a COVID-19 vaccine was considerably higher among respondents sampled in LMICs (mean = 80.3%) when compared to respondents in the US (64.6%) and Russia (30.4%) (33). Similarly, respondents sampled in LMICs tend to view the vaccines as effective and safe more often than respondents in the US or Russia. Several studies reaffirm the widespread willingness across LMICs to vaccinate against COVID-19 (34, 35), despite the lack of access to these vaccines (22, 36–39). Future mixed-method studies should aim to describe the impact of limited access among willing populations and measure any change in willingness due to potential mistrust, which may have developed after such blatant disparities in vaccine distribution.

Despite the general willingness to get vaccinated among those living in LMICs, the coverage of vaccination remains low and some disparities exist among vaccine-hesitant groups compared to those who are willing (4). This lack of vaccine coverage demonstrated a diminished impact on the deaths averted among LIC and LMICs in comparison to HICs. The impact of vaccination was the lowest among low-income countries, with 26.23 deaths averted per 10,000 vaccines, compared to 46.14 deaths averted per 10,000 vaccines in high-income countries (4). The diminished impact among low-income countries was largely attributed to low vaccination coverage (4). Our study demonstrated that low-income countries on average had to wait longer for vaccine rollout and this delay was attributed to an increase in cumulative cases. An increase in cumulative cases would place an undue strain on already fragile healthcare and economic systems. For every additional day until the first vaccine rollout, LICs, on average, reported a 1.1% increase in cumulative cases. Interestingly, UMICs saw almost double the increase in cases for every additional day; however, on average, UMICs waited much less days than LICs until the first vaccine rollout. Therefore, the delayed access to vaccines among non-HICs may be attributed to increasing case counts in the first half of the COVID-19 pandemic.

Continued transmission and circulation of COVID-19 in largely susceptible populations could also increase the likelihood of new variants emerging (40). Our study demonstrated that delays in vaccination among LICs, LMICs, and UMICs were associated with an increase in cumulative cases, possibly signaling a local epidemic that was still growing in size rather than tapering. In a separate multi-strain, metapopulation model, greater vaccine inequities between high-income and low- or lower-middle-income countries led to subsequent global peaks that were larger in size and arrived sooner (40). Vaccine sharing that is equitable across all countries could reduce the susceptible population acutely, therefore limiting the potential for variant emergence.

### 4.1. Strengths and limitations

To the authors' knowledge, this study is among the first to examine economic factors at the country level and their association with COVID-19 outcomes using real-world data. Previous studies primarily relied on modeling outcomes, which are largely limited to the assumptions and parametrization of those models, obscuring our ability to gain a comprehensive understanding of the impact. Our study adds relevant context to the literature by reflecting on and providing a comprehensive and thorough assessment of what is taking place on the grounds due to delays in access.

While ecological studies typically warrant caution in their inference when applied to causal theories at the individual level, this study explored the timing of access to vaccines at the country level and how these delays were modified by a country's economic standing. Therefore, an ecological study would be appropriate for our question. That being said, there may exist within-country inequities of vaccine distribution across local cities or territories that are not captured in this aggregated view. Future studies should aim to further disaggregate available data, to capture within-country inequities of vaccine distribution, which would aid in country-level pandemic preparedness and response efforts. Limited data availability and data quality also presented some limitations to the scope of our analysis. As seen with other studies, which aimed to measure the impact of COVID-19 vaccines, new variants, unreliability in the accuracy of reported data (i.e., death tolls), limited and varied testing protocols and reporting capacity, varied test-seeking behaviors between countries, and varied vaccine rollout strategies complicated attempts to precisely measure the overall impact (4). Efforts to identify the needs of individual countries to support data tracking, capture, and timely reporting are essential, as many pandemic initiatives rely on data for prioritization, identifying inequities, and quantifying impact.

## 5. Conclusion

In this study, we found an average of a 100-day delay to the first COVID-19 vaccination in low-income countries compared to high-income countries. Additionally, among low-income countries, these delays were associated with an increase in cumulative COVID-19 cases. If equitable vaccine distribution and efforts to increase vaccine uptake were prioritized at the beginning of the pandemic, there may have been fewer cumulative cases and associated deaths. Accurately assessing outcomes (i.e., cumulative cases, excess deaths, etc.) is essential to designing and evaluating public health initiatives, quantifying disease burden and disparities, and identifying resource and system needs to support the equitable allocation of resources. Additionally, the use of real-world data provides an accurate estimation of the impact of delays in access, setting the stage for larger conversations and studies to inform policies that prioritize all countries for global allocation of vaccines, instead of perpetuating a bidding war that centers on countries with the highest economic power. Our study illuminates the impact of delayed access, which is essential for policy and program development and implementation for the future pandemic response, especially as new variants emerge that differ in transmissibility, virulence, and/or resistance. Future studies should examine the impact that vaccine rollouts had on country-level transmission dynamics, assessing how delays in vaccination affected the growth phase between each country. Additionally, future studies should examine the impact of misinformation and disinformation on pandemic response, pandemic preparedness, and find solutions to mitigate widespread beliefs surrounding and influencing vaccine hesitancy.

## Data availability statement

The original contributions presented in the study are included in the article/Supplementary material, further inquiries can be directed to the corresponding authors.

## Author contributions

BD and NK conceived the study with input from RL. NK led the data curation and analysis. BD and NK produced the first draft of the manuscript and have accessed and verified the underlying data. All authors read, contributed to, and approved the final draft. All authors had full access to all data included in the study, had equal and final responsibility for the decision to submit for publication, and agreed to be accountable for the content of the work.
