# Supplementary material for: The impact of delayed access to COVID-19 vaccines in low- and lower-middle-income countries
Source: Front Public Health. 2023 Jan 12;10:1087138. doi: 10.3389/fpubh.2022.1087138 (PMC9878283; doi:10.3389/fpubh.2022.1087138)
Supplement: Supplementary file 1 [file Data_Sheet_1.docx]

Supplementary Material

**Table S1.** Data sources

| **Data Description** | **Source** | **URL** |
| --- | --- | --- |
| COVID-19 Deaths, Case Counts, Vaccination Rates, Date of First Vaccine, and First Vaccine Authorization | World Health Organization | <https://covid19.who.int/data> |
| Income Categorization | World Bank | <http://databank.worldbank.org/data/download/site-content/CLASS.xlsx> |
| Population Size | World Bank | <https://data.worldbank.org/indicator/SP.POP.TOTL> |
| Testing Data | Our World in Data | <https://github.com/owid/covid-19-data/tree/master/public/data> |


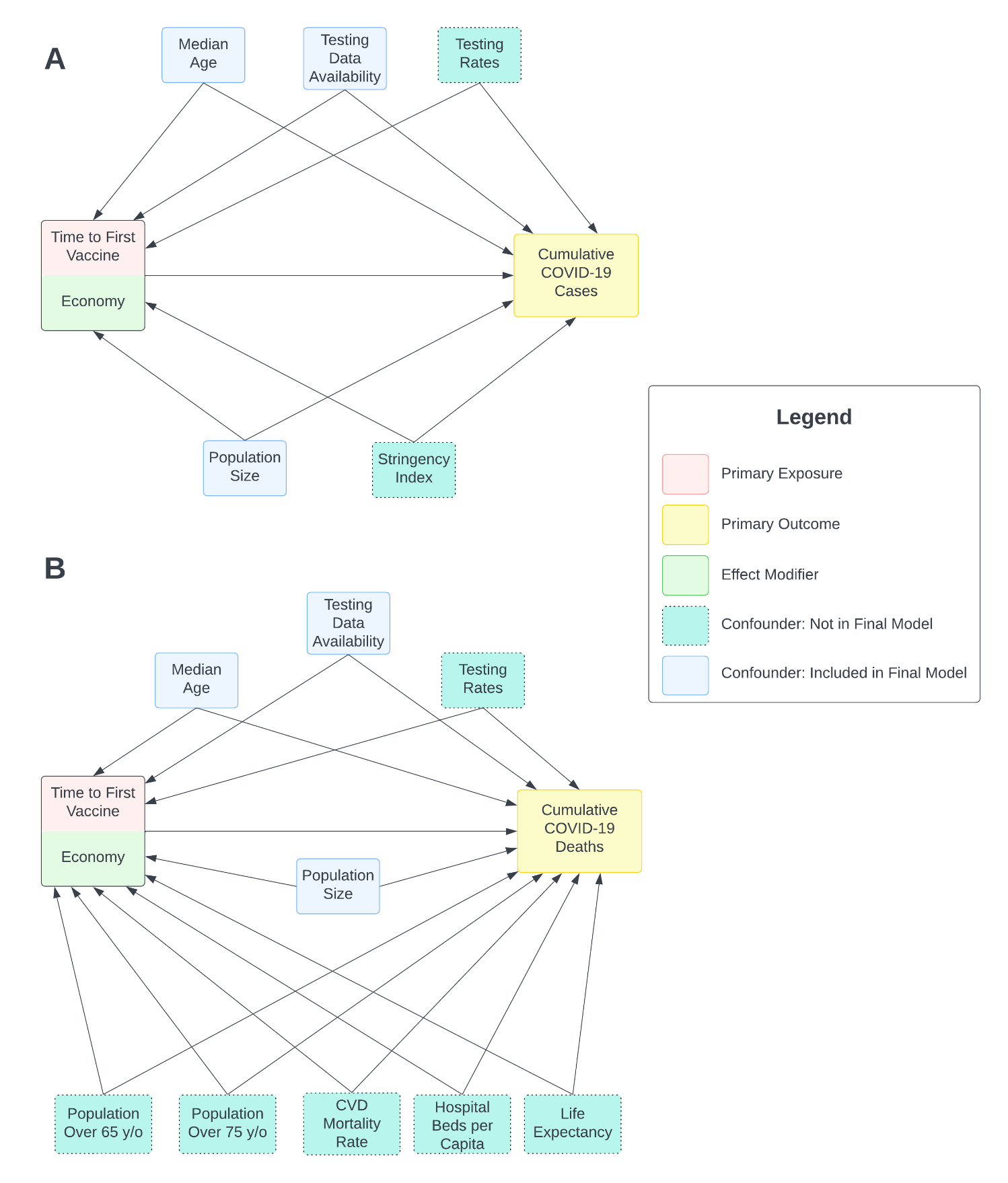


**Figure S1**. Directed acyclic graphs (DAGs) representing the hypothesized ecological causal relationship between time to first vaccine and COVID-19 cases and deaths.

**Table S2**. Comparing COVID-19 Testing Data Availability by Economic Classifications. Odds ratios (OR) were estimated using simple logistic regression, in which the odds of no data availability were modeled as the outcome.

| **Economic Classification** | **Testing Data Availability** | | | |
| --- | --- | --- | --- | --- |
|  | **N** | **Not Available**  **n (%)** | **Available**  **n (%)** | **OR (95% CI)**  **p-value*** |
| Overall |  |  |  |  |
| World Bank Income Classification | 170 | 77 | 93 |  |
| Low Income | 23 | 17 (73.91) | 6 (26.09) | 9.917 (3.202, 30.711)  < 0.0001 |
| Lower Middle Income | 51 | 31 (60.78) | 20 (39.22) | 5.425 (2.31, 12.730)  0.0001 |
| Upper Middle Income | 42 | 17 (40.48) | 25 (59.52) | 2.380 (0.978, 5.793)  0.0561 |
| High Income | 54 | 12 (22.22) | 42 (77.78) | *Reference* |
| GDP PPP Classification | 168 | 75 | 93 |  |
| <7.47 | 19 | 14 (73.68) | 5 (26.32) | 15.862 (3.214, 78.288)  0.0007 |
| 7.78-8.48 | 29 | 21 (72.41) | 8 (27.59) | 14.870 (3.409, 64.857)  0.0003 |
| 8.49-9.47 | 48 | 23 (47.92) | 25 (52.08) | 5.212 (1.349, 20.135)  0.0167 |
| 9.48-10.46 | 52 | 14 (26.92) | 38 (73.08) | 2.087 (0.529, 8.227)  0.2931 |
| >10.46 | 20 | 3 (15.00) | 17 (85.00) | *Reference* |
| GDP PPP (Continuous) |  |  |  |  |
| Mean (95% CI) |  | 24.155  (23.363, 24.947) | 26.299  (25.711, 26.888) | -2.144 (-3.095, -1.194) **  <0.001 *** |

* P-value for beta coefficient

** Mean difference between groups and 95% CI

*** P-value for t-test with equal variance (Equality of Variances: p = 0.0725).

**Table S3**. Comparing mean time to first vaccine (days) by World Bank Income category (F_3,164_ = 32.58, p <0.001). Cells represent mean difference between indicated groups and 95% confidence intervals on the second row.

| **World Bank Income Category** | **Lower middle income** | **Upper middle income** | **High income** |
| --- | --- | --- | --- |
| Low income | 32.756  (-6.17, 71.682) | 54.316  (14.153, 94.48) * | 89.533  (50.829, 128.238) * |
| Lower middle income |  | 21.56  (-10.238, 53.359) | 56.778  (26.843, 86.712) * |
| Upper middle income |  |  | 35.217  (3.691, 66.744) * |

* p < 0.001 (Bonferroni-corrected)

**Table S4**. Comparing mean time to first vaccine (days) by GDP category (F_4,163_ = 26.34, p <0.001). Cells represent mean difference between indicated groups and 95% confidence intervals on the second row.

| **GDP Category** | **7.78 – 8.48** | **8.49 – 9.47** | **9.48 – 10.46** | **> 10.46** |
| --- | --- | --- | --- | --- |
| < 7.47 | 24.822  (-21.221, 70.865) | 48.559  (6.277, 90.842) * | 80.838  (39.019, 122.657) * | 100.034  (50.058, 150.01) * |
| 7.78 – 8.48 |  | 23.737  (-12.953, 60.427) | 56.016  (19.861, 92.17) * | 75.212  (29.87, 120.554) * |
| 8.49 – 9.47 |  |  | 32.279  (1.054, 63.504) * | 51.475  (9.957, 92.993) * |
| 9.48 – 10.46 |  |  |  | 19.196  (-21.85, 60.242) |

* p < 0.001 (Bonferroni-corrected

| **Table S5**. Assessing the interaction between economic classification on the associations between time to first vaccine and cumulative mortality. Modeled using multiple linear regression, p-values for t-test unless otherwise indicated. | | | | | | |
| --- | --- | --- | --- | --- | --- | --- |
| **Parameter** | **Cumulative Deaths** | | | | | |
|  | **Crude Model** | | **Full model without Interaction *** | | **Full Model with Interaction *** | |
|  | **β (95% CI)** | **p-value** | **β (95% CI)** | **p-value** | **β (95% CI)** | **p-value** |
| **I. World Bank Income Classifications** |  |  |  |  |  |  |
| Days to Vaccine | -0.023 (-0.031, -0.015) | <0.0001 | -0.007 (-0.015, 0.001) | 0.0718 | -0.019 (-0.037, -0.002) | 0.0336 |
| Income Classifications |  |  |  |  |  |  |
| Low Income |  |  | -0.79 (-2.426, 0.847) | 0.342 | -1.492 (-11.108, 8.124) | 0.7596 |
| Lower Middle Income |  |  | -0.277 (-1.463, 0.908) | 0.6448 | -2.504 (-11.319, 6.312) | 0.5755 |
| Upper Middle Income |  |  | 0.697 (-0.196, 1.59) | 0.125 | -9.821 (-18.015, -1.628) | 0.0191 |
| High Income |  |  | **—** | **—** | **—** | **—** |
| Income Classifications x Days to Vaccine |  |  |  |  |  |  |
| Low Income |  |  |  |  | 0.004 (-0.019, 0.026) | 0.7541 |
| Lower Middle Income |  |  |  |  | 0.007 (-0.016, 0.029) | 0.5627 |
| Upper Middle Income |  |  |  |  | 0.027 (0.006, 0.048) | 0.0134 |
| High Income |  |  |  |  | **—** | **—** |
| **II. GDP PPP** |  |  |  |  |  |  |
| Days to Vaccine |  |  | -0.007 (-0.015, 0.001) | 0.0681 | -0.079 (-0.147, -0.011) | 0.0239 |
| GDP PPP |  |  | 0.109 (-0.429, 0.647) | 0.6889 | -1.072 (-2.314, 0.17) | 0.0901 |
| GDP PPP x Days to Vaccine |  |  |  |  | 0.003 (0, 0.005) | 0.0392 |
| **III. GDP PPP per Capita** |  |  |  |  |  |  |
| Days to Vaccine |  |  | -0.008 (-0.016, -0.001) | 0.0336 | -0.024 (-0.052, 0.004) | 0.0942 |
| GDP** |  |  |  |  |  |  |
| < 7.47 |  |  | 0.568 (-1.089, 2.224) | 0.4996 | -1.881 (-14.469, 10.708) | 0.7683 |
| 7.78 – 8.48 |  |  | 1.117 (-0.319, 2.554) | 0.1265 | 2.062 (-11.348, 15.472) | 0.7617 |
| 8.49 – 9.47 |  |  | 1.308 (0.189, 2.427) | 0.0223 | -7.088 (-19.308, 5.133) | 0.2537 |
| 9.48 – 10.46 |  |  | 1.096 (0.147, 2.045) | 0.0239 | -8.219 (-19.485, 3.047) | 0.1516 |
| > 10.46 (Reference) |  |  | **—** | **—** | **—** | **—** |
| Days to Vaccine x GDP** |  |  |  |  |  |  |
| < 7.73 |  |  |  |  | 0.009 (-0.023, 0.041) | 0.5871 |
| 7.74 – 8.97 |  |  |  |  | 0.001 (-0.034, 0.035) | 0.9754 |
| 8.98 – 10.21 |  |  |  |  | 0.022 (-0.01, 0.054) | 0.1745 |
| 9.48 – 10.46 |  |  |  |  | 0.025 (-0.005, 0.055) | 0.1067 |
| > 10.21 (Reference) |  |  | — | — | — | — |

GDP = Gross domestic product; PPP = Purchasing power parity

*Adjusted for 2021 population size, median age, and testing data availability (yes/no)

**Log-transformed


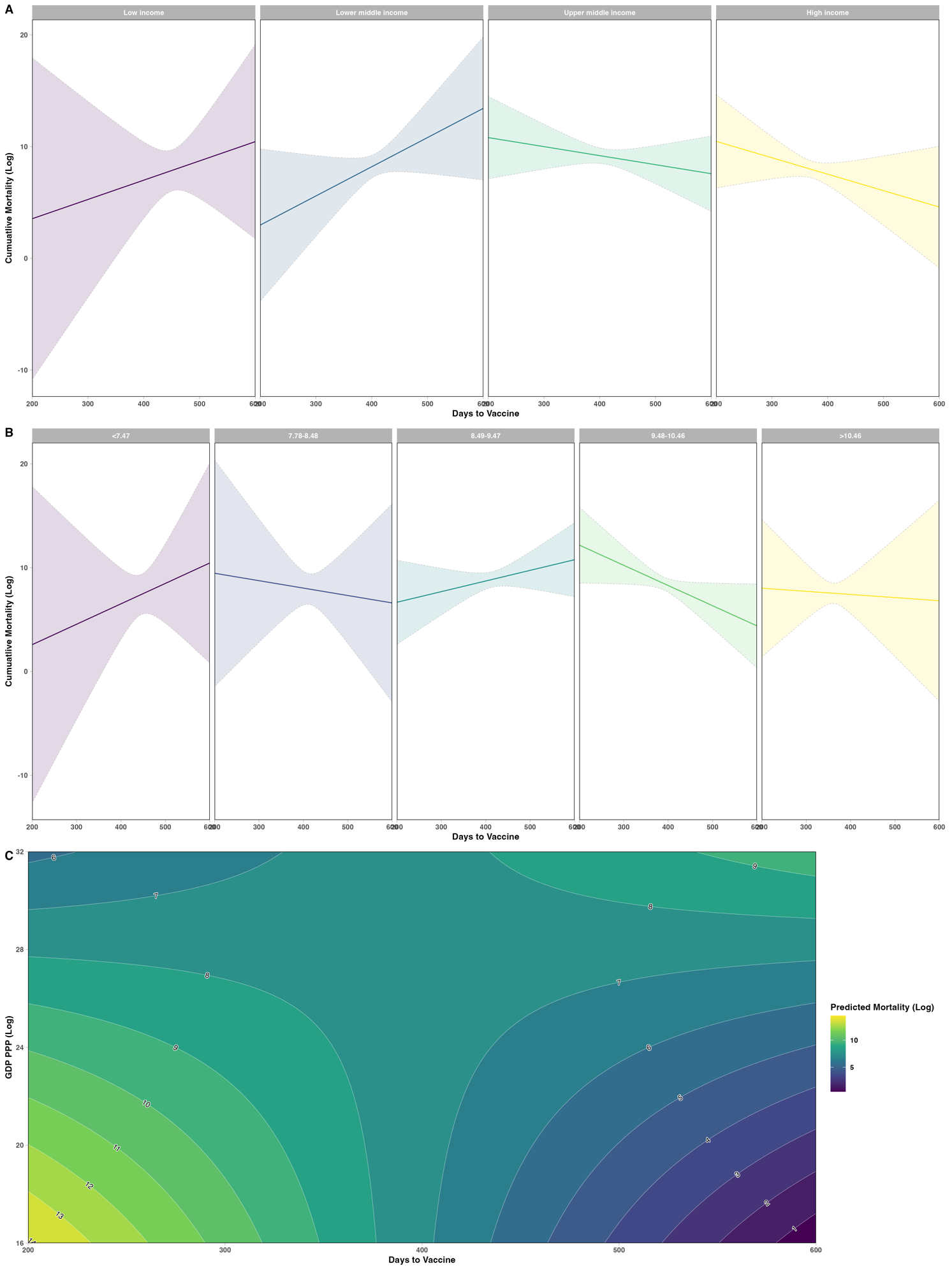


**Figure S2**. Assessing the interaction between economic size and days to first COVID-19 vaccine on estimated log-cumulative mortality. Adjusted for 2021 population size, median age, and testing data availability (yes/no). Shaded bands indicate 95% prediction intervals.


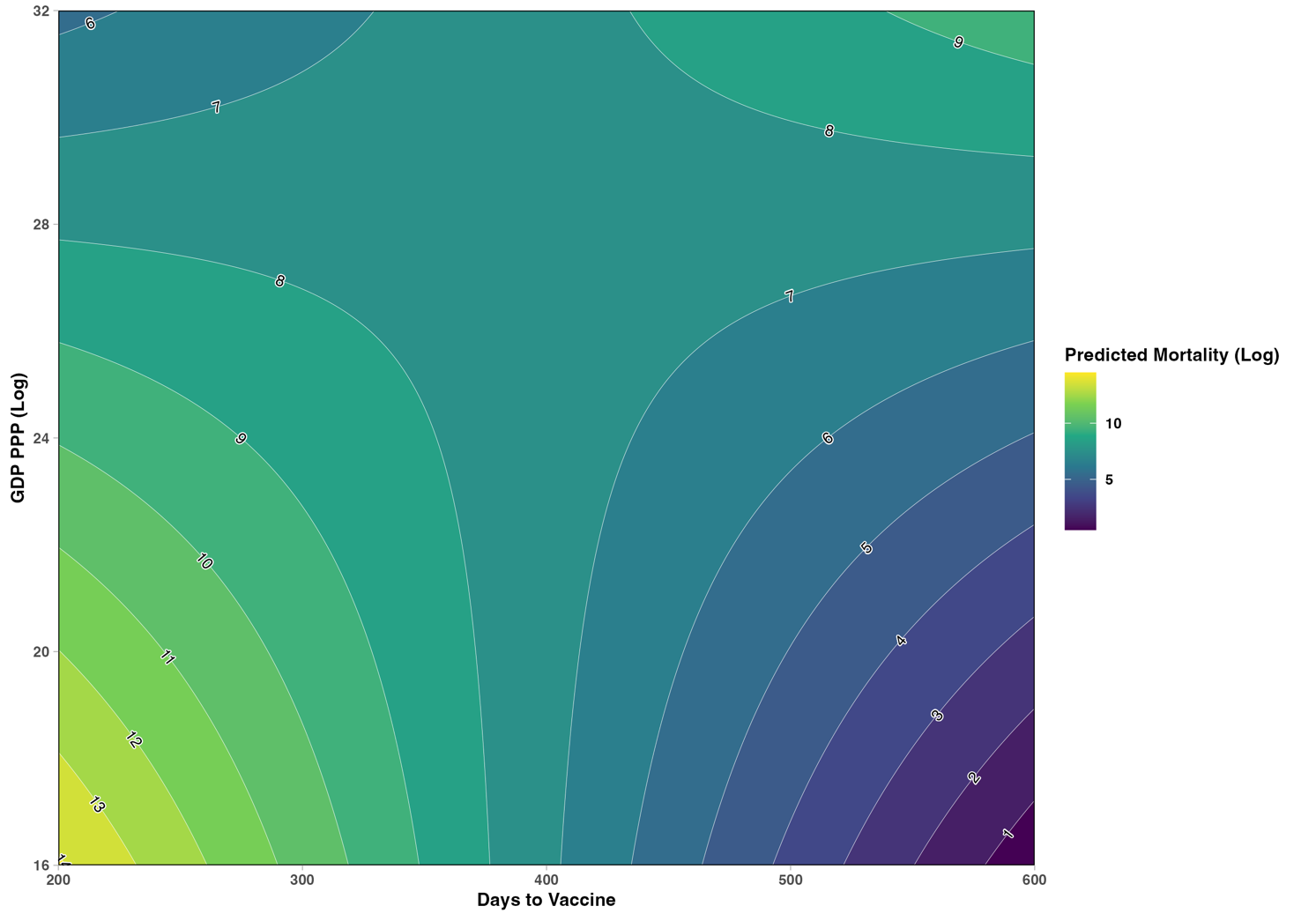


**Figure S3**. Contour plot demonstrating the interaction between log-GDP PPP and days to first COVID-19 vaccine on cumulative mortality estimated using multiple linear regression and adjusted for 2021 population size, median age, and testing data availability (yes/no).

| **Table S6**. Assessing the interaction between economic classification on the associations between time to first vaccine and cumulative cases. Models are limited to countries with testing data availability and modeled using multiple linear regression. P-values for t-test unless otherwise indicated. | | | | | | |
| --- | --- | --- | --- | --- | --- | --- |
| **Parameter** | **Cumulative Cases** | | | | | |
|  | **Crude Model** | | **Full model without Interaction *** | | **Full Model with Interaction *** | |
|  | **β (95% CI)** | **p-value** | **β (95% CI)** | **p-value** | **β (95% CI)** | **p-value** |
| **I. World Bank Income Classifications** |  |  |  |  |  |  |
| Days to Vaccine | -0.013 (-0.023, -0.002) | 0.0173 | -0.007 (-0.017, 0.003) | 0.1454 | -0.017 (-0.036, 0.002) | 0.0739 |
| Income Classifications |  |  |  |  |  |  |
| Low Income |  |  | 1.081 (-0.858, 3.02) | 0.2709 | -9.693 (-31.382, 11.996) | 0.3766 |
| Lower Middle Income |  |  | 0.976 (-0.254, 2.206) | 0.1184 | -9.035 (-22.521, 4.452) | 0.1863 |
| Upper Middle Income |  |  | 1.39 (0.458, 2.321) | 0.0039 | -2.39 (-11.685, 6.905) | 0.6104 |
| High Income |  |  | — | — | — | — |
| Income Classifications x Days to Vaccine |  |  |  |  |  |  |
| Low Income |  |  |  |  | 0.025 (-0.024, 0.074) | 0.3082 |
| Lower Middle Income |  |  |  |  | 0.025 (-0.009, 0.059) | 0.1405 |
| Upper Middle Income |  |  |  |  | 0.01 (-0.014, 0.034) | 0.4072 |
| High Income |  |  |  |  | — | — |
| **II. GDP PPP** |  |  |  |  |  |  |
| Days to Vaccine |  |  | -0.004 (-0.014, 0.005) | 0.3709 | -0.048 (-0.176, 0.08) | 0.4602 |
| GDP PPP |  |  | -0.483 (-1.117, 0.152) | 0.1339 | -1.149 (-3.205, 0.907) | 0.2697 |
| GDP PPP x Days to Vaccine |  |  |  |  | 0.002 (-0.003, 0.007) | 0.5001 |
| **III. GDP PPP per Capita** |  |  |  |  |  |  |
| Days to Vaccine |  |  | -0.006 (-0.015, 0.004) | 0.2188 | -0.017 (-0.05, 0.015) | 0.2955 |
| GDP** |  |  |  |  |  |  |
| < 7.47 |  |  | 0.838 (-1.138, 2.814) | 0.4014 | -10.693 (-35.781, 14.396) | 0.3989 |
| 7.78 – 8.48 |  |  | 0.82 (-0.768, 2.408) | 0.3072 | -2.12 (-23.114, 18.874) | 0.8412 |
| 8.49 – 9.47 |  |  | 1.445 (0.31, 2.579) | 0.0132 | -6.196 (-19.937, 7.545) | 0.3722 |
| 9.48 – 10.46 |  |  | 0.966 (0.138, 1.794) | 0.0228 | -0.497 (-13.737, 12.744) | 0.9407 |
| > 10.46 (Reference) |  |  | — | — | — | — |
| Days to Vaccine x GDP** |  |  |  |  |  |  |
| < 7.73 |  |  |  |  | 0.027 (-0.032, 0.086) | 0.3638 |
| 7.74 – 8.97 |  |  |  |  | 0.008 (-0.045, 0.061) | 0.7689 |
| 8.98 – 10.21 |  |  |  |  | 0.02 (-0.017, 0.056) | 0.2921 |
| 9.48 – 10.46 |  |  |  |  | 0.004 (-0.032, 0.041) | 0.8113 |
| > 10.21 (Reference) |  |  |  |  | — | — |

GDP = Gross domestic product; PPP = Purchasing power parity

*Adjusted for 2021 population size, median age, and testing rates

**Log-transformed

| **Table S7**. Assessing the interaction between economic classification on the associations between time to first vaccine and cumulative deaths. Models are limited to countries with testing data availability and modeled using multiple linear regression. P-values for t-test unless otherwise indicated. | | | | | | |
| --- | --- | --- | --- | --- | --- | --- |
| **Parameter** | **Cumulative Deaths** | | | | | |
|  | **Crude Model** | | **Full model without Interaction *** | | **Full Model with Interaction *** | |
|  | **β (95% CI)** | **p-value** | **β (95% CI)** | **p-value** | **β (95% CI)** | **p-value** |
| **I. World Bank Income Classifications** |  |  |  |  |  |  |
| Days to Vaccine | -0.011 (-0.024, 0.001) | 0.0773 | -0.004 (-0.016, 0.009) | 0.556 | -0.015 (-0.038, 0.009) | 0.221 |
| Income Classifications |  |  |  |  |  |  |
| Low Income |  |  | 0.549 (-1.904, 3.002) | 0.6577 | -13.327 (-40.431, 13.777) | 0.3309 |
| Lower Middle Income |  |  | 0.678 (-0.878, 2.234) | 0.3888 | -15.673 (-32.527, 1.181) | 0.0679 |
| Upper Middle Income |  |  | 1.399 (0.22, 2.578) | 0.0206 | -0.993 (-12.609, 10.623) | 0.8654 |
| High Income |  |  | — | — | — | — |
| Income Classifications x Days to Vaccine |  |  |  |  |  |  |
| Low Income |  |  |  |  | 0.032 (-0.029, 0.093) | 0.3009 |
| Lower Middle Income |  |  |  |  | 0.041 (-0.001, 0.083) | 0.0575 |
| Upper Middle Income |  |  |  |  | 0.007 (-0.023, 0.037) | 0.6605 |
| High Income |  |  |  |  | — | — |
| **II. GDP PPP** |  |  |  |  |  |  |
| Days to Vaccine |  |  | -0.001 (-0.013, 0.011) | 0.8322 | -0.001 (-0.162, 0.16) | 0.9907 |
| GDP PPP |  |  | -0.579 (-1.373, 0.215) | 0.1505 | -0.574 (-3.154, 2.006) | 0.6592 |
| GDP PPP x Days to Vaccine |  |  |  |  | 0 (-0.006, 0.006) | 0.9968 |
| **III. GDP PPP per Capita** |  |  |  |  |  |  |
| Days to Vaccine |  |  | -0.003 (-0.015, 0.009) | 0.5886 | -0.003 (-0.043, 0.037) | 0.8824 |
| GDP* |  |  |  |  |  |  |
| < 7.47 |  |  | 0.925 (-1.542, 3.393) | 0.4578 | -9.973 (-40.821, 20.876) | 0.5218 |
| 7.78 – 8.48 |  |  | 1.158 (-0.824, 3.141) | 0.2486 | 2.263 (-23.551, 28.077) | 0.8619 |
| 8.49 – 9.47 |  |  | 1.803 (0.386, 3.22) | 0.0132 | -4.005 (-20.901, 12.89) | 0.6384 |
| 9.48 – 10.46 |  |  | 1.231 (0.197, 2.265) | 0.0202 | 7.432 (-8.849, 23.712) | 0.3664 |
| > 10.46 (Reference) |  |  | — | — | — | — |
| Days to Vaccine x GDP* |  |  |  |  |  |  |
| < 7.73 |  |  |  |  | 0.023 (-0.05, 0.095) | 0.537 |
| 7.74 – 8.97 |  |  |  |  | -0.004 (-0.069, 0.061) | 0.9003 |
| 8.98 – 10.21 |  |  |  |  | 0.013 (-0.032, 0.058) | 0.5604 |
| 9.48 – 10.46 |  |  |  |  | -0.016 (-0.061, 0.028) | 0.4654 |
| > 10.21 (Reference) |  |  |  |  | — | — |

GDP = Gross domestic product; PPP = Purchasing power parity

*Adjusted for 2021 population size, median age, and testing rates

**Log-transformed
